# Ameliorative effects of Kyung-Ok-Ko and its mixture with *Pueraria lobata* Ohwi on postmenopausal osteoporosis by promoting phytoestrogenic activity in rats

**DOI:** 10.3389/fnut.2023.1171346

**Published:** 2023-06-26

**Authors:** Minseo Kim, Hyun-Sook Kim, Joohee Oh, Xiangqin Zhou, SongHee Ahn, Youngtae Koo, Hyun-Jung Kim, Jiwon Jang

**Affiliations:** ^1^Department of Food and Nutrition, Sookmyung Women’s University, Seoul, Republic of Korea; ^2^Natural Products Convergence R&D Division, Kwangdong Pharm Co., Ltd., Seoul, Republic of Korea

**Keywords:** Kyung-Ok-Ko, *Pueraria lobata* Ohwi, phytoestrogen, ovariectomy, menopause, osteoporosis, autophagy, postmenopausal syndrome

## Abstract

**Introduction:**

Kyung-Ok-Ko (KOK) is a popular traditional medicine used as a natural alternative to hormone replacement therapy for treating postmenopausal symptoms in Asia. *Pueraria lobata* Ohwi (*P. lobata*) is rich in isoflavones and has been traditionally used in combination with other herbs to produce synergistic and pharmaceutical effects *via* a multi-target approach for disease treatment. We aimed to investigate the phytoestrogenic effects of KOK extract against postmenopausal symptoms in ovariectomized (OVX) rats and confirm its efficacy by mixing KOK and *P. lobata* extracts.

**Methods:**

OVX rats were daily oral administrated with KOK and KOK + *P. lobata* mixture extracts (300–400 mg/kg) and their body weight and tail temperature were monitored for 12 weeks. The biochemical parameters, estradiol levels, and bone turnover markers were measured in the serum samples. Moreover, the estrogen receptor, ER-α and ER-β expression in the uterus and the uterus morphology were evaluated. AMPK, ATG1/ULK1, and mTOR protein expression in the liver were assessed.

**Results:**

The 12-week treatment with KOK and KOK + *P. lobata* mixture extracts did not cause liver damage or hormonal changes in the OVX rats. The treatments reduced the high lipid accumulation-related body weight gain and the tail temperature increase that was induced by ovariectomy. Further, it exhibited protective effects against hyperlipidemia and osteoporosis. No significant difference was observed in uterine weight compared to the OVX-treated group, while endometrial thickness reduction inhibition was observed due to ovariectomy. Bone mineral density (BMD) and serum osteocalcin levels, which decreased in OVX rats, increased with both treatments. Western blotting analysis showed that ER-α and ER-β were not expressed in the treated rats, whereas these proteins were expressed in Sham-operated rats. No significant differences in the phosphorylation of AMPK were observed; however, the ATG1/ULK1 and mTOR protein phosphorylation levels were upregulated and downregulated in the treated rats compared to those of OVX rats, respectively.

**Conclusion:**

This is the first *in vivo* study observing the efficacy and synergistic effects of the mixture of KOK and *P. lobata*. Our results suggest the potential of KOK and KOK + *P. lobata* mixture as an alternative therapy for alleviating menopausal symptoms.

## Introduction

The postmenopausal period accounts for over one-third of a woman’s life expectancy, and the aging population is increasing (1). Menopause is a biological stage in which menstruation stops due to deficiency of estrogen, a hormone that prevents ovarian failure and bone loss in women. The symptoms of women with menopause include facial flushing, anxiety, depression, vaginal dryness, sleep disorders, and joint pain due to the discontinuation of estrogen production in the ovaries; moreover, they are at risk for chronic diseases such as dyslipidemia and osteoporosis (1, 2).

Osteoporosis is typically a senile disease emerging as a public health issue due to the increased life expectancy and aging population. It is a metabolic bone disease caused by the decrease in bone mineral density (BMD), damage to the bone microstructure, and weakening of bone strength, and characterized by an increased possibility of fracture (3). In the elderly, osteoporosis fracture is one of the main causes of death, especially in postmenopausal women, who have a greater risk of osteoporosis fractures than men; therefore, its management is crucial (4, 5).

Postmenopausal osteoporosis, caused by estrogen deficiency, leads to an increase in the bone replacement rate due to an imbalance between osteoblastic and osteoclastic activities. A decrease in endogenous estrogen production during menopause results in a significant increase in bone turnover, increasing the risk of bone loss and fractures (6). Estrogen was previously shown to prevent apoptosis in osteocytes by regulating autophagy (7). Estrogen deficiency increased osteoclastogenesis, osteoclast lifespan, and bone turnover rates, causing greater resorption than formation (8). A recent study showed that estrogen deficiency inhibited autophagy and promoted apoptosis in alveolar-process osteocytes. Although the pathogenesis of osteoporosis is multifactorial, the primary biological mechanism of bone mass loss is a tilt in the bone formation–absorption balance toward osteoclasts. For alleviating postmenopausal osteoporosis, estrogen replacement therapy can enhance osteocyte viability by inhibiting apoptosis and maintaining autophagy (9).

Hormone replacement therapy (HRT) using estrogen and progesterone has been used for its management till date. However, according to the 2002 Women’s Health-led Study (WHI) of 16,608 menopausal women aged 50–79 years, hormone therapy with estrogen and progesterone increased the incidence of breast cancer and cardiovascular disease by 26 and 29%, respectively, compared to those of the control group (10). Because of the high risk of the chronic use of HRT, phytoestrogens have emerged as an alternative for managing the symptoms of menopause.

Phytoestrogens such as ginsenoside and resveratrol are structurally similar to estrogen. They can combine with estrogen receptors (ERs) to exert a multitude of benefits for which estrogen is responsible, and have attracted interest as potential bone health therapies in estrogen-deficient postmenopausal women (11). It has been a logical and valuable treatment option to use phytoestrogens in the management of postmenopausal osteoporosis. Studies have shown that phytoestrogens can reduce the risk of fractures in postmenopausal women by preserving BMD and decreasing the rate of bone loss (12). In the last few decades, numerous *in vitro*, *in vivo*, and clinical studies have evaluated the therapeutic effects of phytoestrogens on bone health, though the effect of phytoestrogens on postmenopausal osteoporosis remains unclear. A previous study showed that phytoestrogens exerted their bone protective effect by inhibiting bone resorption and enhancing bone formation (13).

Kyung-Ok-Ko (KOK) is a traditional oriental medicine consisting of four ingredients*: Panax ginseng, Wolfiporia extensa, Rehmannia glutinosa*, and honey. In East Asia, KOK has long been administered as a vitalizing medicine for healthy people, or with medicinal intent to treat patients with various age-related symptoms. Phytochemical and pharmacological investigations have shown that KOK possesses a variety of bioactivities, including anti-fatigue, anti-inflammatory, immune-enhancing, antioxidant, and weight loss activities (14–18). Currently, the Ministry of Food and Drug Safety of South Korea has registered its efficacy as a nutritional tonic for treating post-illness weakness, physical fatigue, malaise, and menopausal disorders (Registration No. 196300084). *Panax ginseng* acts as a phytoestrogen *via* ginsenosides Rg1, Rb2, and Re (6, 19). AMP-activated protein kinase (AMPK) and Autophagy-related gene 1 (ATG1)/Unc-51-like kinase 1 (ULK1) — a critical metabolic sensor that upregulates autophagy (20).

The substances in *Wolfiporia extensa* are known to significantly reduce the plasma calcitonin gene-related peptide concentration and menopausal hot flushes (MHF) (21). In addition, KOK has a proliferative effect on osteoblasts without *in vitro* cytotoxic effects, decreases the bone loss induced by lipopolysaccharide, prevents estrogen deficiency-induced bone loss, inhibits the deterioration of the trabecular microarchitecture, decreases the number of T lymphocytes, maintains the bone turnover rate, and inhibits uterine weight reduction *in vivo* (22, 23).

*Pueraria lobata* Ohwi (*P. lobata*) contains high levels of isoflavones and has been traditionally used in combination with other herbs to produce synergistic and pharmacological effects *via* a multi-target approach for disease treatment (24). It has various effects that alleviate cardiovascular diseases, liver diseases, and osteoporosis (25). The main active component of *P. lobata*, puerarin, prevented osteoporosis in ovariectomized (OVX) animal models by regulating the autophagy inhibitor mammalian target of rapamycin (mTOR) (24, 26, 27).

Many studies have shown that continuous supplementation of KOK and *P. lobata* is expected to alleviate menopausal symptoms and postmenopausal osteoporosis, respectively (14–18, 24, 26, 27). However, until now, no studies have been conducted to reveal the mechanism or confirm the efficacy of combining KOK and *P. lobata*. We hypothesized that KOK and *P. lobata* extracts could alleviate postmenopausal osteoporosis by stimulating autophagy-activating kinases, such as AMPK and ATG1/ULK1, and regulating mTOR levels. We investigated whether KOK and a KOK + *P. lobata* mixture are beneficial treatments for menopause-induced osteoporosis using an OVX rat model. Further, we determined whether 12 weeks of continuous administration affected liver function and the estrogen levels.

The aim of this article is to give an overview of the bidirectional interrelationship between postmenopausal osteoporosis and KOK or the KOK + *P. lobata* mixture, identify the bone metabolic effects of these phytoestrogens, and report their potential bone protective effects in postmenopausal osteoporosis model.

## Materials and methods

### Preparation of sample extracts

KOK and *P. lobata* were standardized and provided by Kwangdong Pharm Co., Ltd. (Seoul, Republic of Korea). The provided KOK was prepared by adding *Panax ginseng* powder (6.2 g), *Wolfiporia extensa* (12.4 g), and honey (41.9 g) to the juice obtained by adhering *Rehmannia glutinosa* (39.9 g), and after 120 h of aging at 94°C, d-sorbitol was added and the mixture was cooled to 35°C (15). *P. lobata* was extracted using 50% ethanol, concentrated, dried, and mixed with the KOK extract. The mixture quality and homogeneity were confirmed based on product standards and test methods. The 17β-estradiol was purchased from Sigma-Aldrich Chemical Co. (St. Louis, MO, USA). In determining the daily oral dose of 12 weeks, this study followed previous studies of KOK and *P. lobata*, and other herbal extract supplementation studies (11, 14–19). Also, OECD guidelines recommend a limit of 1,000 mg/kg/day for repeated oral supplementation tests in rats (28), so 300 mg/kg, 350 mg/kg, and 400 mg/kg of KOK and KOK + *P. lobata* mixture extracts were administered. The dose of 17β-estradiol was according to previous studies. 17β-estradiol is known that 2,000 to 10,000 mg/kg in oral administration in rats were considered lethal dose for 50 percent (LD50), and there have been previous studies administrated 1 mg/kg as a positive control in the OVX rat model (29, 30). There were no animals with death or clinically abnormal reactions observed during the 12-week oral administration period.

### Experimental animals and treatments

**Figure 1:**
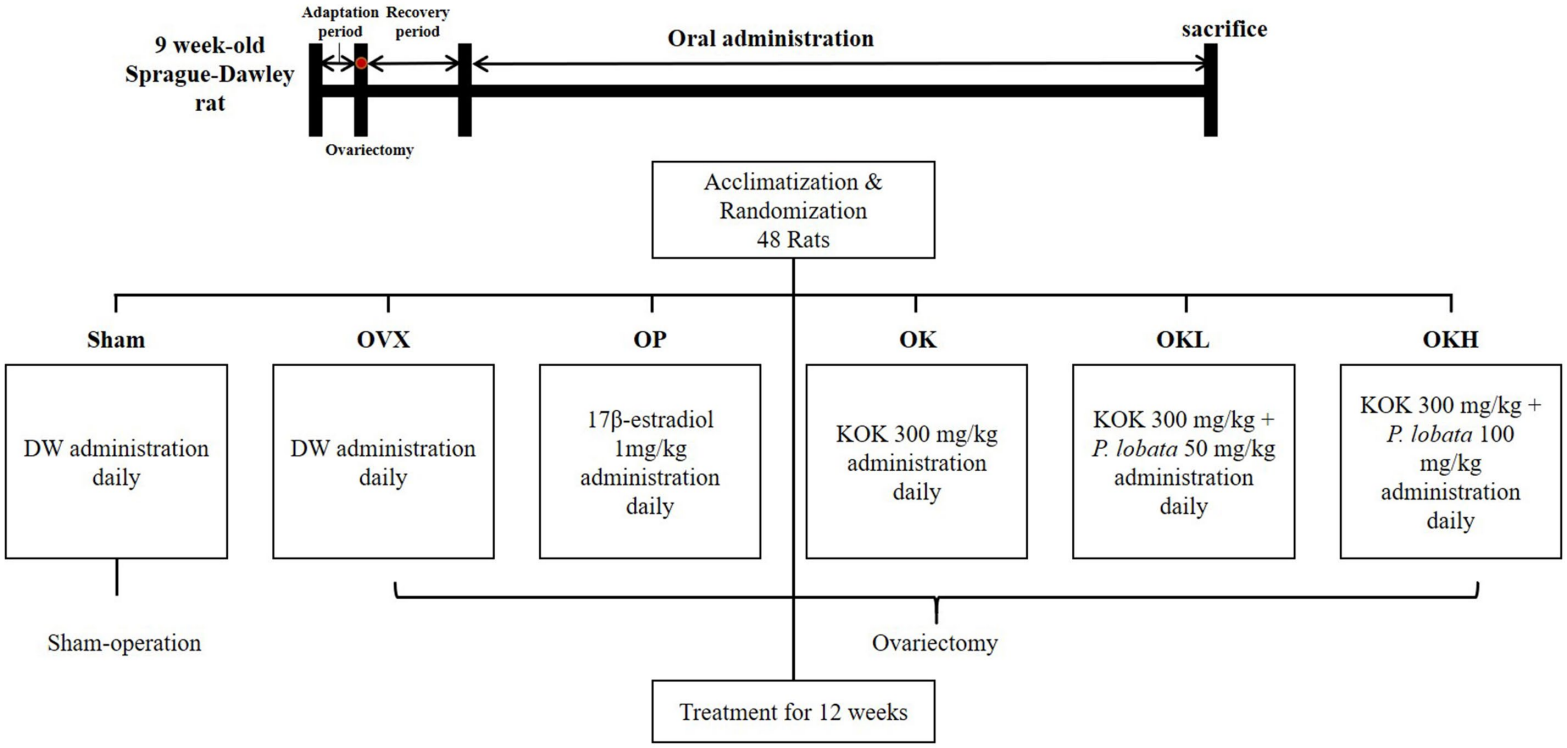
Flow chart of the randomization and treatment schedule.

Forty-eight 9 week-old female Sprague (SD) rats, weighing 170–175 g, were obtained from Saeronbio, Gyeonggi-do, Korea. The animals were housed in a standard metal cage (3 per cage) under controlled temperature (21 ± 1°C), and humidity (50–60%) conditions, and 12-h dark/light cycles throughout the study. The rats had *ad libitum* access to water and food. A week after adaptation, bilateral ovariectomy (*n* = 40) and Sham operations (*n* = 8) were performed through an incision in the back under general anesthesia with an intraperitoneal injection of Zoletil50 (Virbac, Carros, France) and Rompun (Bayer Healthcare Korea, Seoul, Republic of Korea) in a 1:1 mixture at a dose of 0.10–0.13 ml. Approximately 1.5 cm of the skin, abdominal cavity, and muscles were incised and the ovaries were exposed. In each ovariectomy, the oviduct was ligated with a black silk thread bilaterally, whereas the remaining eight animals underwent Sham surgery, in which the bilateral ovaries were examined and returned to the original position under the same protocol. After 2 weeks of recovery, the rats were divided into six randomized groups among which one was a Sham-operated group and five were OVX groups (Figure 1). Each group had *n* = 8 rats and the following groups were formed: (1) Sham-operated control group (Sham); (2) OVX control group (OVX); (3) OVX + positive control group (OP); (4) OVX + KOK (OK); (5) OVX + KOK + *P. lobata* low dose (OKL); (6) OVX + KOK + *P. lobata* high dose (OKH). All groups were treated with oral gavage administration of distilled water (Sham, OVX), 1 mg/kg 17β-estradiol (OP), 300 mg/kg KOK (OK), 350 mg/kg KOK and *P. lobata* mixture (OKL), and 400 mg/kg KOK and *P. lobata* mixture (OKH) for 12 weeks. The determination of the dose was based on the dose that showed anti-inflammatory, anti-fatigue, and anti-immune effects in previous studies on KOK and *P. lobata*. For administrating 300 mg/kg, it is equivalent to administrating 48 mg/kg per day for an adult of 60 kg (15). All experimental procedures were approved by the Animal Ethics Committee of the Sookmyung Women’s University for the Care and Use of Laboratory Animals (SMWU-IACUC-2102126). The study was conducted in compliance with all relevant guidelines, including the ARRIVE guidelines.

Oral administration of treatment was performed 7 days a week at 10:00 a.m., and clinical indicators were observed daily. Food intake was measured three times a week, the tail temperature was measured twice a week, and the weight was measured once a week.

After 12 weeks of oral administration, rats were fasted for 12 h and euthanized using CO_2_ gas. The serum samples were collected and centrifuged at 3,000 rpm and stored at −80°C for biochemical analyses. Liver, uterus, femur, and abdominal fat tissues were collected and weighed.

### Tail temperature measurements

The tail temperature was measured using an infrared thermometer (153-IRB, BiosebLab, Paris, France) at the tail, 2 cm from the rectum. It was recorded at a certain time twice a week in stable conditions for the experimental animals.

### Biochemical parameters

Serum concentrations were collected through cardiac puncture using needles and centrifuged at 3,000 rpm, 4°C for 30 min (Combi-450R, Hanil Co. Ltd., Seoul, Korea). To assess the lipid marker status of each group, the serum triglyceride (TG), total cholesterol (TC), and high-density lipoprotein cholesterol (HDL-C) levels were measured using a TG-S kit (AM157S, Asanpharm, Hwaseong, Korea), a T-CHO kit (AM202, Asanpharm, Hwaseong, Korea), and HDL-CHO kits (AM203, Asanpharm, Hwaseong, Korea), respectively. The serum low-density lipoprotein cholesterol (LDL-C) and very-low-density lipoprotein cholesterol (VLDL-C) levels were calculated using the Friedwald method (31). Hepatic profiles were determined based on the aspartate aminotransferase (AST) and alanine aminotransferase (ALT) levels using AST (AM103-K, Asanpharm, Hwaseong, Korea) and ALT assay kits (AM102, Asanpharm, Hwaseong, Korea). Serum estradiol (E2) levels were measured using an estradiol kit (ES180S-100, CalBiotech, Spring Valley, CA, USA). The bone formation marker osteocalcin level was determined using a rat osteocalcin kit (NBP2-68153, Novus, USA). All enzyme-linked immunosorbent assay kits were used according to the manufacturer’s instructions.

### Histopathology

To measure the endometrial length of the uteri, samples were fixed in a 10% neutral buffered formalin solution, processed, and embedded in paraffin. The tissues were sliced into sections using a rotary microtome and subjected to hematoxylin and eosin (H&E) staining. Uterine tissue slides were examined and photographed under an Olympus light microscope (BX43F, Tokyo, Japan) at 100× magnification to determine endometrium thickness.

### Dual energy X-ray absorptiometry analysis of femur

To evaluate the BMD, femur samples were placed in a 10% neutral buffered formaldehyde solution and a dual-energy X-ray bone densitometer (PIXIMUS, Lunar Corp, USA) was used.

### Western blotting

To examine the effect of KOK and *P. lobata* on menopause-induced osteoporosis, the uterine and liver tissues of 48 rats (*n* = 8, 6 groups) were used. The samples were extracted by homogenizing 0.01 ± 0.002 mg of the liver and uterine tissues using a Pro-Prep kit (17,081, iNtRON Biotechnology, Gyeonggi-do, Korea).

Protein concentration was determined using a bicinchoninic acid protein analysis assay. Proteins (25 μg) were separated *via* SDS-PAGE using 8% gels and transferred onto polyvinylidene fluoride membranes (Merck Millipore, MA, United States). The membranes were blocked in 5% BSA for 1 h, at 4°C, followed by overnight incubation at 4°C with primary antibodies against ER-α (1:1000; Cat#L0821), ER-β (1:1000; Cat#G0621), AMPK (1:500; Cat#2532S), p-AMPK (1:1000; Cat#2531S), mTOR (1:1000; Cat#2972S), p-mTOR (1:1000; Cat#2971S), ULK1 (1:1000; Cat#8054S), p-ULK1 (1:1000; Cat#14202S), and GAPDH (1:5000; Cat#5174S). Immunoreactive band intensities were quantified using densitometric analysis (Biomolecular Imaging System-3; Ge Healthcare, United States).

### Statistical analysis

All data were expressed as mean ± standard deviation. Statistical analyses were performed using SPSS software, version 25 (SPSS, Inc., Chicago, IL, USA). Two-way ANOVA analysis of variance followed by Duncan’s multiple range test was used to assess differences between groups. A value of *p* < 0.05 was considered statistically significant.

## Results

### Effects of KOK and *Pueraria lobata* on the body weight and body weight gain

**Figure 2:**
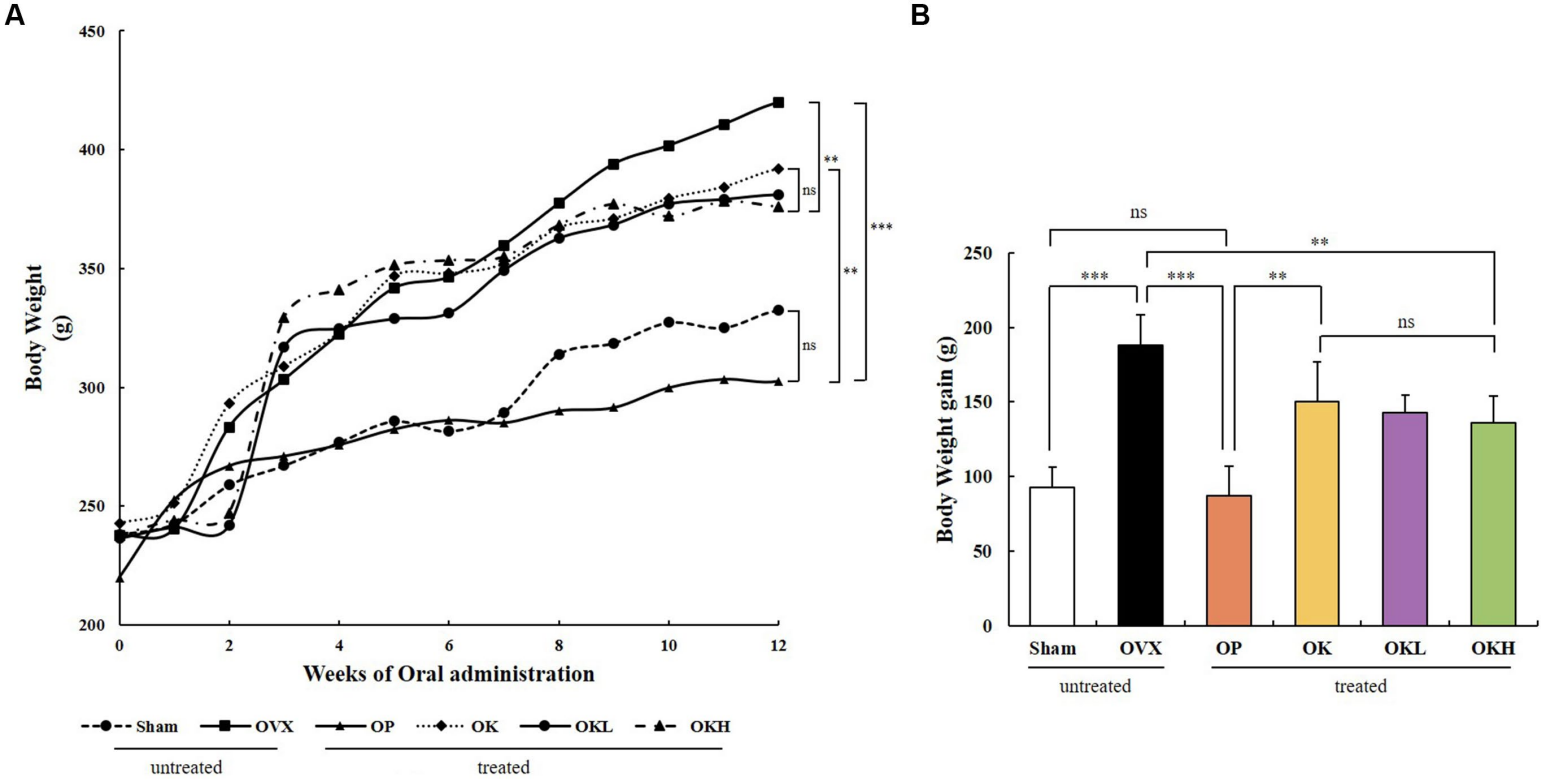
Effects of KOK and KOK + *P. lobata* mixture on body weight. **(A)** Changes in body weight during 12 weeks of oral administration, and **(B)** Final body weight gain. Data represent the mean ± standard error of mean (SEM). “ns” indicates non-significant differences (*p* > 0.05). **p < 0.01; ***p < 0.001 as determined by Duncan’s multiple range test.

Before ovariectomy, there were no significant differences between the body weights of the experimental groups. After surgery and recovery, the 12-week oral administration was initiated. The OVX, OP, OK, OKL, and OKH groups showed a significant increase in body weight and body weight gain throughout the experimental period compared with those of the Sham group. OVX rats had the highest final weight, followed by the OK, OKL, and OKH groups (Figures 2A,B); however, the groups that were administered KOK and *P. lobata* tended to gain less weight as the concentration increased.

### Effects of KOK and *Pueraria lobata* on the uterus weight, coefficient, and endometrial thickness

**Figure 3:**
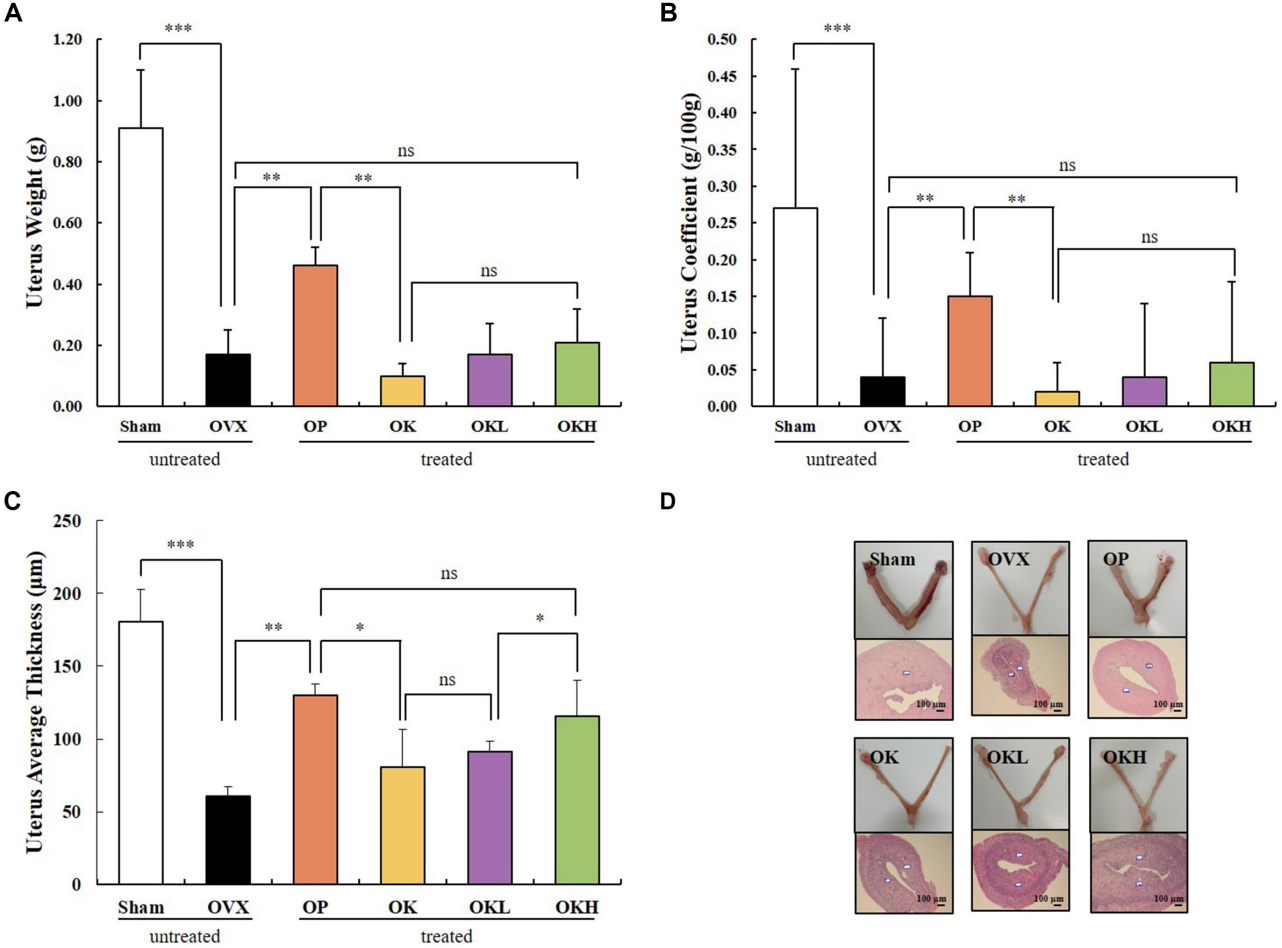
Effects of KOK and KOK + *P. lobata* mixture on uterus. **(A)** Uterus weight, **(B)** Uterus coefficient, **(C)** Uterus average thickness, and **(D)** H&E-stained endometrial sections. Data are represented as the mean ± standard error of mean (SEM). “ns” indicates non-significant differences (*p* > 0.05). *p < 0.05; **p < 0.01; ***p < 0.001 as determined by Duncan’s multiple range test.

Uterine weight, uterine coefficient, and endometrial thickness were measured to confirm the effect of ovariectomy and the efficacy of continuous administration of *KOK* and *P. lobata*. As shown in Figures 3A,B, the uterine weight and coefficient values of the OVX rats were significantly lower than those of the Sham group rats. However, the administration of KOK and KOK + *P. lobata* inhibited further reduction in the average uterine thickness induced by ovariectomy. Groups OK, OKL, and OKH showed a dose-dependent endometrial thickness recovery (OK: 80.5 ± 26.2 μm, OKL: 91.3 ± 7.4 μm, OKH: 115.8 ± 24.4 μm; Figure 3C and Table 1). Figure 3D shows the uterine specimens of each group immediately after sacrifice along with the H&E-stained endometrial sections used to assess the histological differences in uterus appearance and endometrial thickness.

**Table 1:** Effects of KOK and *P. lobata* on the uterus weight, coefficient, and average thickness.

Group	Uterus weight (g)	Uterus coefficient (g/100 g)	Uterus average thickness (μm)
Sham	0.91 ± 0.19	0.27 ± 0.05	180.3 ± 22.3
OVX	0.17 ± 0.08	0.04 ± 0.02	60.8 ± 6.7
OP	0.46 ± 0.06***	0.15 ± 0.02***	130.0 ± 17.7
OK	0.10 ± 0.04	0.02 ± 0.01	80.5 ± 26.2
OKL	0.17 ± 0.10***	0.04 ± 0.02***	91.3 ± 7.4
OKH	0.21 ± 0.11	0.06 ± 0.03	115.8 ± 24.4

### Effects of KOK and *Pueraria lobata* on flushing

**Figure 4:**
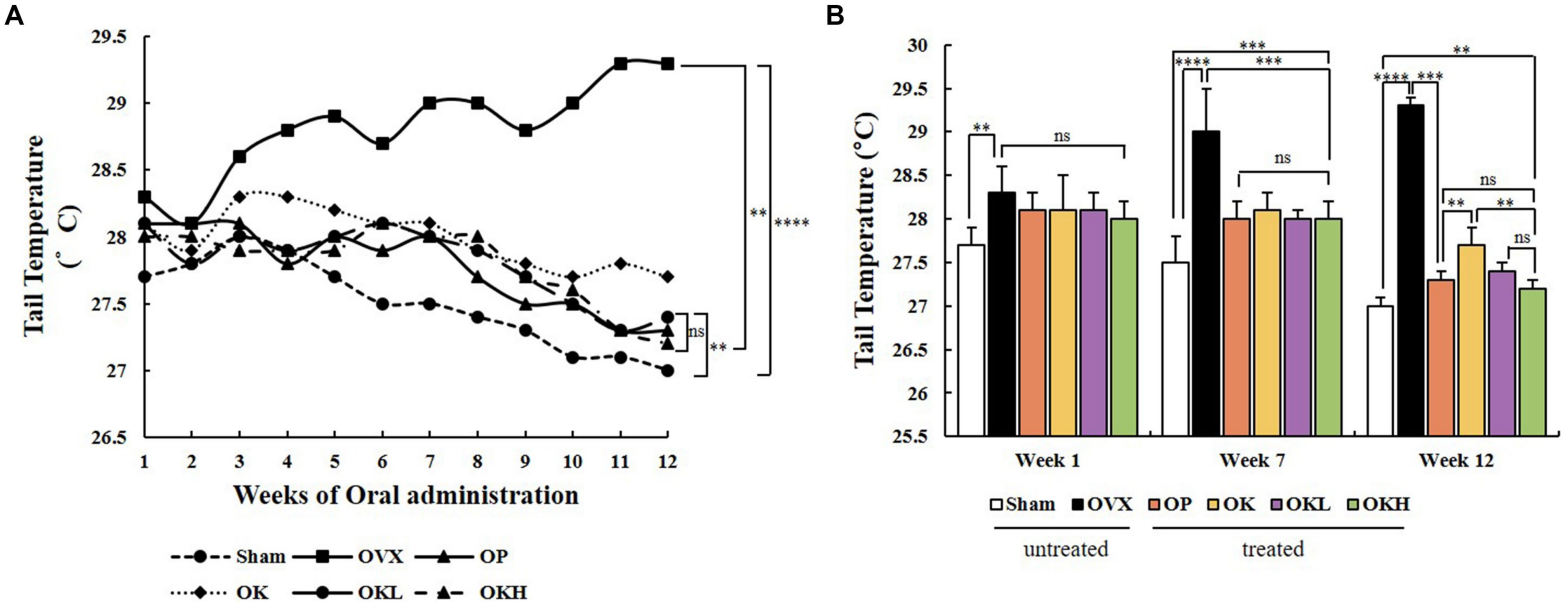
Effects of KOK and KOK + *P. lobata* mixture on flushing response *via* tail temperature measurement. **(A)** Changes in tail temperature during 12 weeks of oral administration, and **(B)** Tail temperatures at week 1, week 7, and week 12. Data represent the mean ± standard error of mean (SEM). “ns” indicates non-significant differences (*p* > 0.05). **p < 0.01; ***p < 0.001; ****p < 0.0001 as determined by Duncan’s multiple range test.

During the oral administration period, the tail temperature was recorded to confirm the alleviating effect on hot flushes. The tail temperature of each experimental animal was measured twice a week and is shown in Figure 4A. According to previous studies, it was reported that the skin temperature increased from the 1st week after ovariectomy (32). Tail temperature was measured upon oral administration from the 3rd week after ovariectomy, and the tail temperature measurement was marked from 1 to 12 weeks according to the oral administration week. In week 1, there was no significant difference in tail temperature among the OVX, OP, OK, OKL, and OKH group rats that had undergone ovariectomy; however, there was a significant difference between that of these and the Sham group rats (*p* < 0.01). At the 7th week of oral administration, the tail temperature of the OP, OK, OKL, and OKH group rats were significantly lower than that of the OVX group (OVX: 29.0 ± 0.5°C, OP: 28.0 ± 0.2°C, OK: 28.1 ± 0.2°C, OKL: 28.0 ± 0.1°C, OKH: 28.0 ± 0.2°C; *p* = 0.000). Finally, in week 12, the tail temperature of the OP, OK, OKL, and OKH group rats were significantly lower than that of the OVX group rats and, in particular, the OKL and OKH groups showed the same level of decrease as the OP group (OP: 27.3 ± 0.1°C, OK: 27.7 ± 0.2°C, OKL: 27.4 ± 0.1°C, OKH: 27.2 ± 0.1°C; *p* = 0.000; Figure 4B). As a result, the final tail temperature decreased by 5.5% for the OK group, 6.5% for the OKL group, and 7.2% for the OKH group compared to that of the OVX group.

### Effects of *KOK* and *Pueraria lobata* on serum biochemistry

#### Lipid profiles

**Figure 5:**
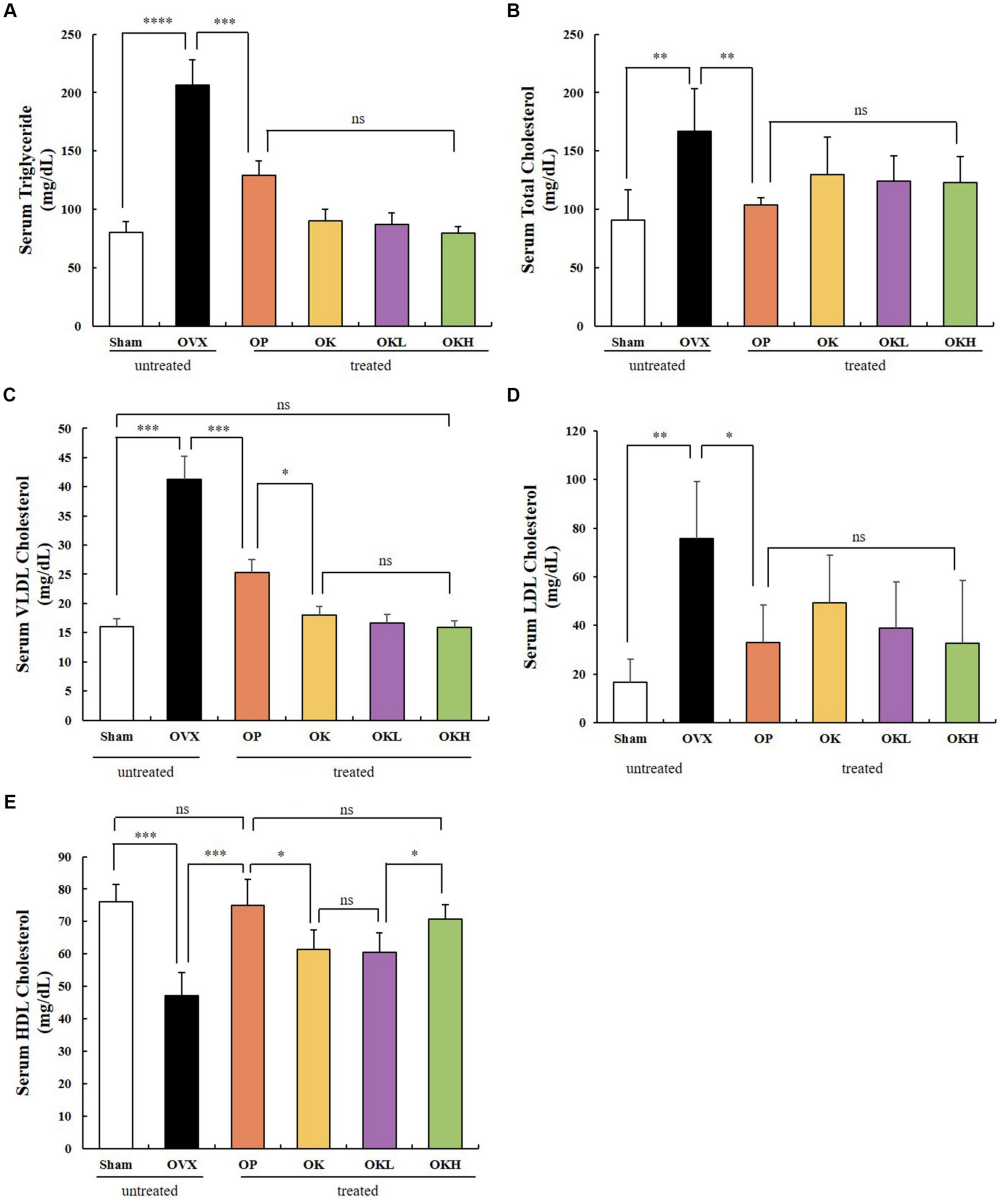
Effects of KOK and KOK + *P. lobata* mixture on serum lipid profiles. **(A)** Serum TG, **(B)** Serum TC, **(C)** Serum VLDL-C, **(D)** Serum LDL-C, and **(E)** Serum HDL-C levels. Data represent the mean ± standard error of mean (SEM). “ns” indicates non-significant differences (*p* > 0.05). *p < 0.05; **p < 0.01; ***p < 0.001; ****p < 0.0001 as determined by Duncan’s multiple range test.

The serum lipid biochemical parameters are summarized in Figure 5 and Table 2. The serum levels of TG, TC, HDL-C, LDL-C, and VLDL-C were measured and calculated to evaluate the increased risk of chronic diseases, such as hyperlipidemia and dyslipidemia, due to decreased estrogen levels after menopause. Except for the OVX group, there were no significant differences in the serum levels of TG, TC, LDL-C, and VLDL-C among the OVX groups (Figures 5A–D). Furthermore, the OK, OKL, and OKH groups showed significantly reduced TG, TC, LDL-C, and VLDL-C levels, similar to the results of the Sham and OP groups (*p* = 0.000). The OVX groups, except for the OKH group (71.6 ± 6.2 mg/dL), showed lower HDL-C levels than the Sham group (OP: 73.1 ± 12.2 mg/dL, OK: 61.1 ± 10.1 mg/dL, OKL: 64.2 ± 17.5 mg/dL); however, all groups showed significantly higher HDL-C values than those of the OVX group (OVX: 46.9 ± 8.7 mg/dL; p = 0.000; Figure 5E).

**Table 2:** Effects of KOK and *P. lobata* on the serum lipid profiles.

Group	TG (mg/dL)	TC (mg/dL)	HDL-C (mg/dL)	LDL-C (mg/dL)	VLDL-C (mg/dL)
Sham	80.5 ± 9.1	91.9 ± 27.7	78.2 ± 12.4	40.9 ± 10.6	15.4 ± 2.7
OVX	206.5 ± 21.6	175.4 ± 58.5	46.9 ± 8.7	109.6 ± 54.0	41.3 ± 4.3
OP	126.3 ± 13.2^###^	104.2 ± 10.4^##^	73.1 ± 12.2^###^	31.6 ± 12.7^###^	25.3 ± 2.6^#^
OK	90.5 ± 9.6	126.3 ± 36.6	61.1 ± 10.1	92.3 ± 41.2	18.1 ± 1.9
OKL	81.9 ± 12.4^###^	129.3 ± 24.6^##^	64.2 ± 17.5^###^	83.6 ± 32.5^###^	16.4 ± 2.5^#^
OKH	78.6 ± 7.4	126.5 ± 28.0	71.6 ± 6.2	68.6 ± 33.7	15.7 ± 1.5

#### Hepatic function and serum estradiol profiles

**Figure 6:**
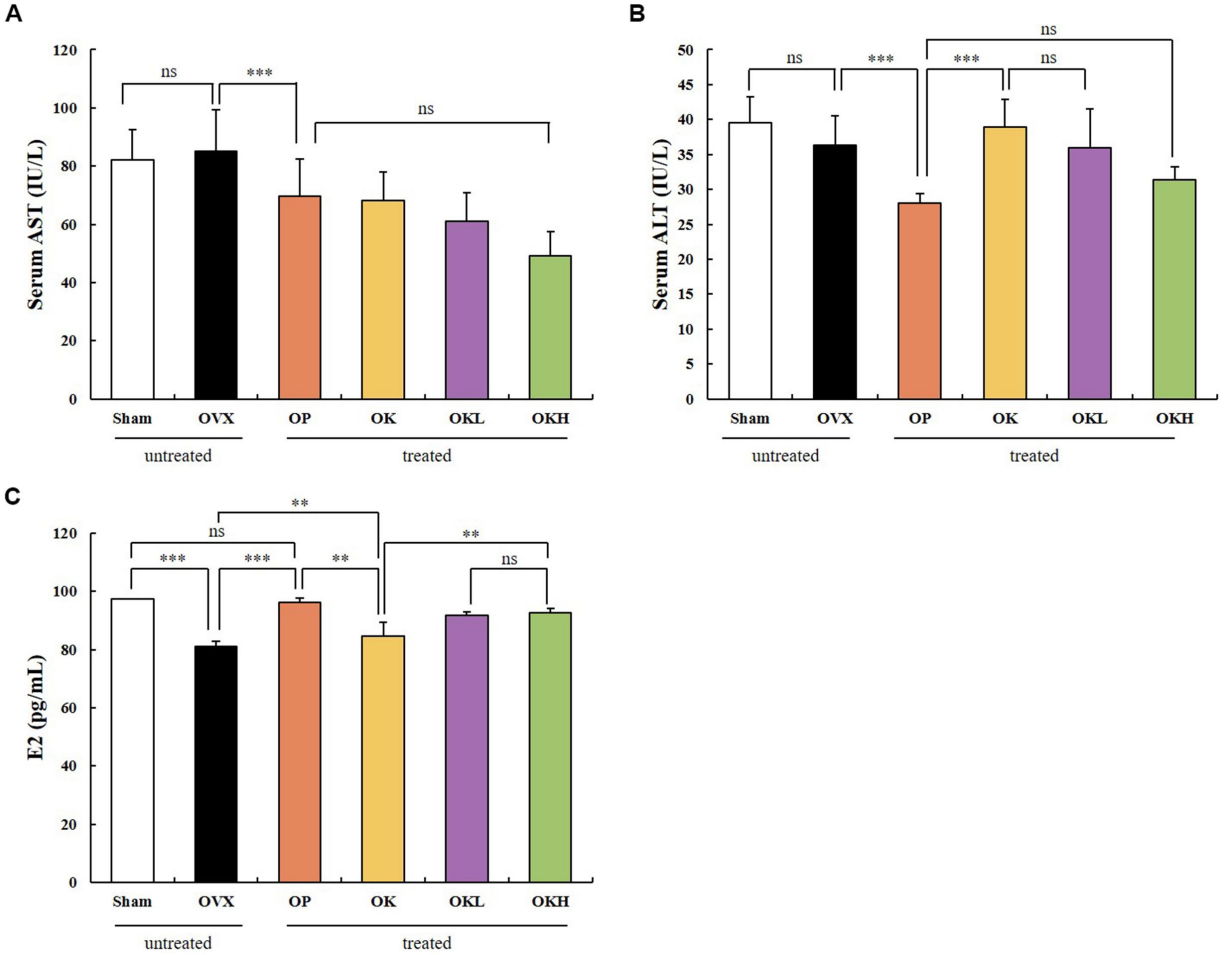
Effects of KOK and KOK + *P. lobata* mixture on serum hepatic function and serum estradiol profiles. **(A)** Serum AST, **(B)** Serum ALT, and **(C)** Serum E2 levels. Data are represented as the mean ± standard error of mean (SEM). “ns” indicates non-significant differences (*p* > 0.05). **p < 0.01; ***p < 0.001 as determined by Duncan’s multiple range test.

The serum hepatic and estradiol profiles are presented in Table 3 and Figure 6. The serum AST and ALT levels were measured to investigate whether the 12-week administration of KOK and *P. lobata* affected liver function and health. Considering that a rat’s normal AST and ALT ranges are 45.7–80.8 IU/L and 17.5–30.2 IU/L, respectively (33), all groups in this experiment were included in the normal range (Figures 6A,B). Accordingly, it was confirmed that the administration of KOK and *P. lobata* did not affect liver function or cause liver damage.

**Table 3:** Effects of KOK and *P. lobata* on serum hepatic function and estradiol profiles.

Group	AST (IU/L)	ALT (IU/L)	E2 (pg/mL)
Sham	82.10 ± 10.30	39.57 ± 3.65	97.26 ± 1.76
OVX	85.09 ± 14.33	36.36 ± 4.15	80.99 ± 1.54
OP	69.77 ± 12.78^###^	28.01 ± 1.34^###^	96.12 ± 4.78
OK	68.03 ± 9.86	38.93 ± 3.96	84.55 ± 1.02
OKL	60.99 ± 9.71	35.98 ± 5.57	91.84 ± 1.49***
OKH	49.08 ± 8.29	31.42 ± 1.85	92.58 ± 1.28

In addition, the serum estradiol (E2) levels were tested to confirm whether the administration of KOK and *P. lobata* for 12 weeks affected the sex hormone levels. We confirmed that the levels of serum E2 were higher in the OK (84.19 ± 1.44 pg./mL), OKL (91.33 ± 3.82 pg./mL), and OKH (92.21 ± 1.38 pg./mL) groups than in the OVX group (81.42 ± 1.66 pg./mL). However, the OP group showed the highest expression of serum E2 (95.16 ± 4.68 pg./mL; Figure 6C). The serum E2 levels vary with the ovulation cycle and compared to previous studies that used the same instrumental method (34); 71–128 pg./mL was observed for non-menstrual or pregnant rats (*n* = 10) and 30.2 ± 2.6 pg./mL to 149.7 ± 11.3 pg./mL was measured in the Sham group according to the hormone cycle (35). Considering the change in estradiol concentration according to the ovulation cycle in previous studies, we confirmed that the administration of KOK and *P. lobata* in this experiment did not affect the E2 levels.

### Bone function and serum osteoblast indicators

**Figure 7:**
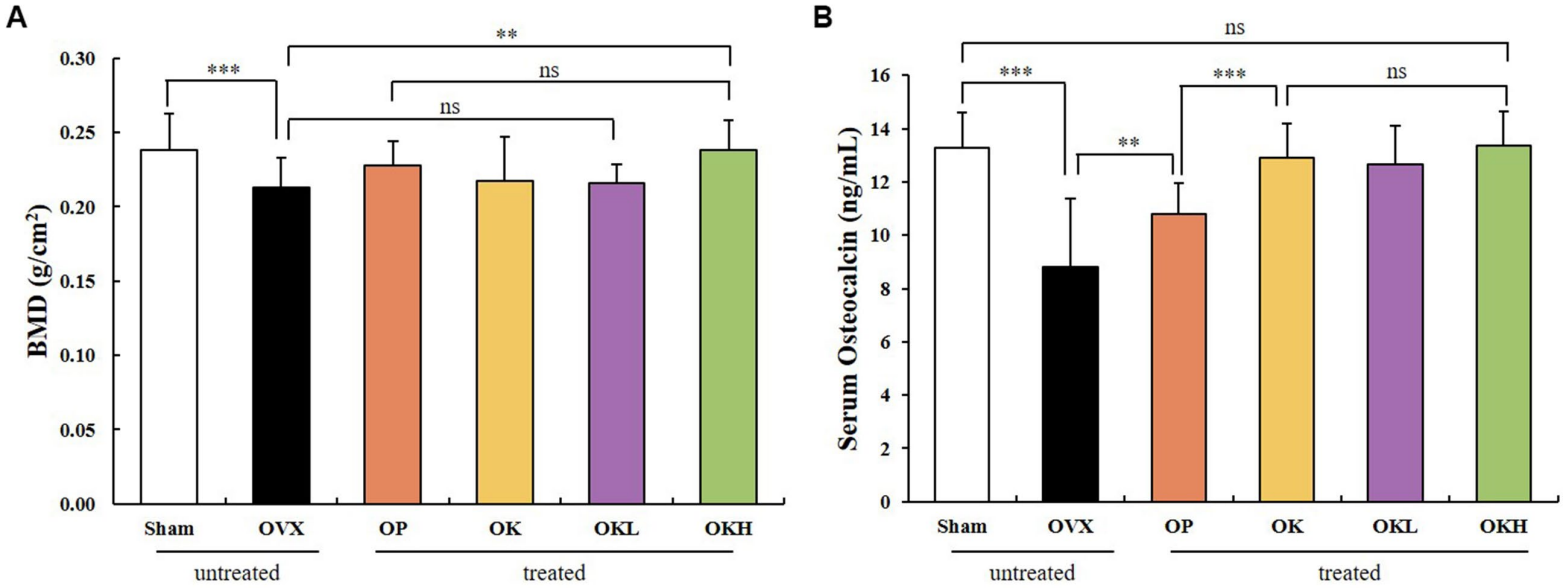
Effects of KOK and KOK + *P. lobata* mixture on BMD of femora and serum osteocalcin indicators. **(A)** BMD of femora, and **(B)** Serum osteocalcin levels. Data are represented as the mean ± standard error of mean (SEM). “ns” indicates non-significant differences (*p* > 0.05). **p < 0.01; ***p < 0.001 as determined by Duncan’s multiple range test.

The BMD of the experimental model was measured using DEXA (Figure 7A). The BMD of the OK (0.22 ± 0.01 g/cm^2^), OKL (0.22 ± 0.02 g/cm^2^), and OKH (0.24 ± 0.02 g/cm^2^) groups tended to increase compared to that of the OVX (0.21 ± 0.02 g/cm^2^) group. Particularly, the BMD of the OKH group was at a similar level to that of the Sham group (0.24 ± 0.02 g/cm^2^).

Serum osteocalcin, a representative marker of osteoblast activation, was measured (Figure 7B). The serum osteocalcin levels in the OK (12.91 ± 1.46 ng/mL), OKL (13.17 ± 1.13 ng/mL), and OKH (13.36 ± 1.16 ng/mL) groups were significantly higher than in the OVX group (8.80 ± 1.18 ng/mL) in a concentration-dependent manner and were similar to those of the Sham group (13.29 ± 2.60 ng/mL).

After testing the BMD and serum osteocalcin levels, it was confirmed that the continuous administration of KOK and *P. lobata* had a positive effect on increasing the BMD and activating the osteoblasts.

### Effects of KOK and *Pueraria lobata* on activating the ER-α and ER-β

**Figure 8:**
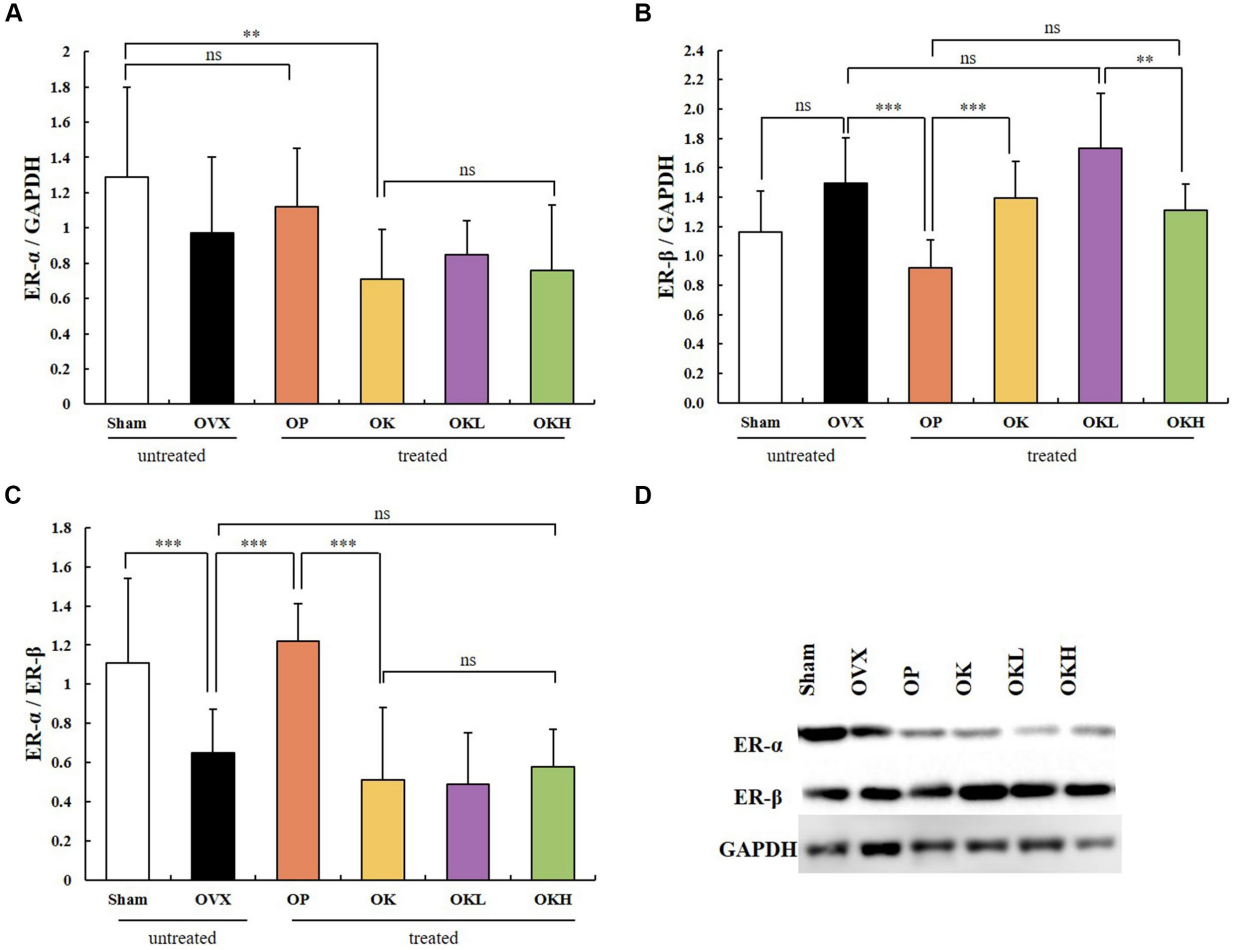
Effects of KOK and KOK + *P. lobata* mixture on western blotting analysis of ER-α and ER-β. **(A)** ER-α/GAPDH, **(B)** ER-β/GAPDH, **(C)** ER-α/ER-β, and **(D)** Representative western blotting of all group showing the ER-α, ER-β, and GAPDH protein levels. Data are represented as the mean ± standard error of mean (SEM). “ns” indicates non-significant differences (*p* > 0.05). **p < 0.01; ***p < 0.001 as determined by Duncan’s multiple range test.

Uterine tissue was extracted and its expression of ER-α and ER-β was measured. In the western blotting assay (Figure 8), no significant differences were observed among the OVX groups (OVX, OP, OK, OKL, and OKH; *p* < 0.01; Figure 8A); however, the expression of ER-β in the OP group was significantly lower than that in the OVX group (*p* < 0.001; Figure 8B). Accordingly, the ER-α to ER-β ratio of the Sham and OP groups was at the same level, and those of the OK, OKL, and OKH groups were relatively lower than that of the OVX group (*p* = 0.000; Figure 8C). As a result, it was confirmed that the 12-week administration of KOK and *P. lobata* did not increase the risk of ER activity, which causes diseases such as breast cancer and uterine myoma in women.

### Effects of KOK and *Pueraria lobata* on activating autophagy by intensifying AMPK and ATG1/ULK1 and inhibiting mTOR

**Figure 9:**
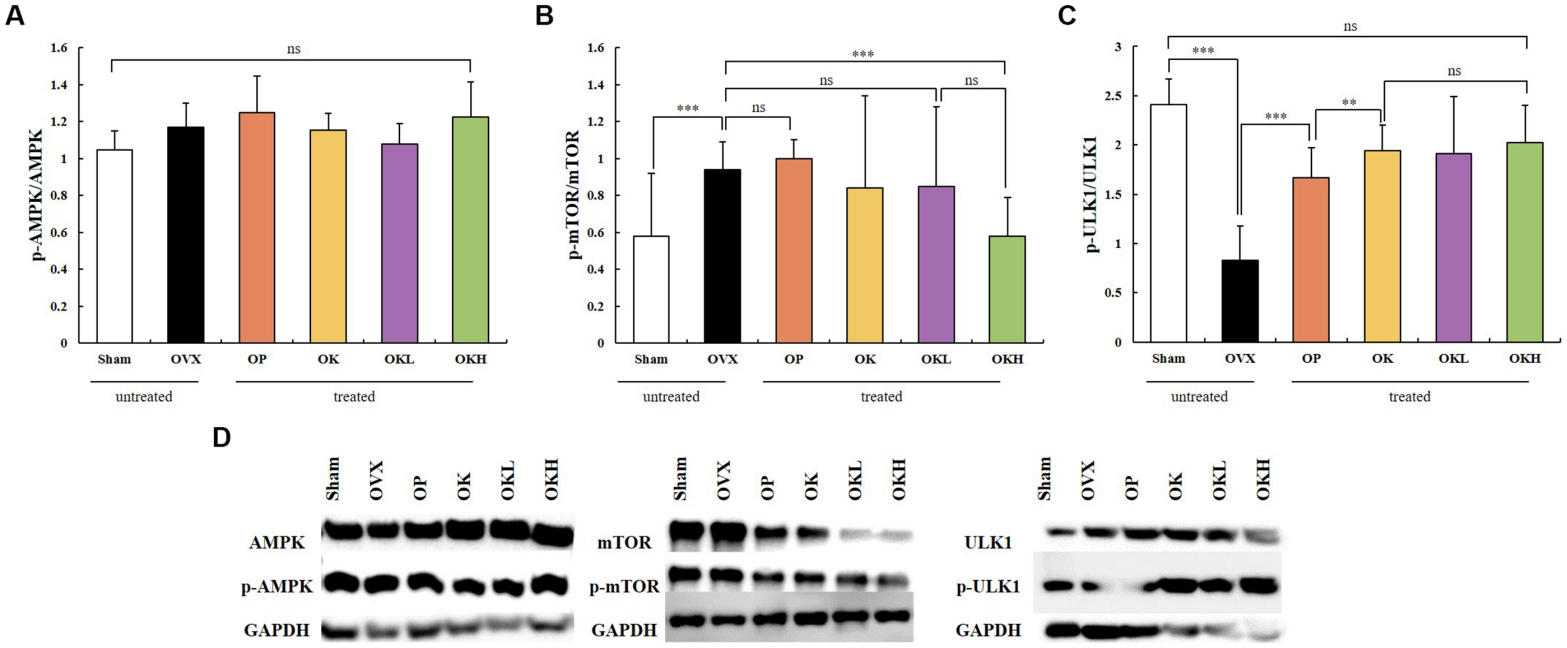
Effects of KOK and KOK + *P. lobata* mixture on western blotting analysis of AMPK, mTOR, ATG1/ULK1 and their phosphorylated forms. **(A)** p-AMPK / AMPK, **(B)** p-mTOR / mTOR, **(C)** p-ULK1 / ULK1, and **(D)** Representative western blotting of all the groups showing AMPK, mTOR, ATG1/ULK1, and their phosphorylated forms, and the GAPDH protein levels. Data are represented as the mean ± standard error of mean (SEM). “ns” indicates non-significant differences (*p* > 0.05). **p < 0.01; ***p < 0.001 as determined by Duncan’s multiple range test.

The expression and activation of AMPK and ATG1/ULK1 proteins, which are involved in activating autophagy, and the mTOR protein, which is involved in inhibiting autophagy, were investigated. A western blotting assay of liver tissues showed that the groups administered KOK and the KOK + *P. lobata* mixture showed an increase in AMPK and ATG1/ULK1 and a decrease in mTOR. The degree to which the AMPK and ULK1 levels increased and the mTOR levels decreased was higher in the OP group than in the Sham group. Subsequently, the activities of AMPK, ULK1, and mTOR were determined based on their degrees of phosphorylation. The phosphorylation of AMPK showed no statistical significance in all groups (Figure 9A). However, compared with those of the OVX group, the phosphorylation of ULK1 was significantly higher in the OK, OKL, and OKH groups (*p* = 0.000; Figure 9C), whereas the phosphorylation of mTOR was significantly lower than the OVX group (p = 0.000; Figure 9B).

## Discussion

Estrogen deficiency in postmenopausal women and OVX rats promotes chronic diseases (36). OVX rats presented weight gain, a tail temperature increase, uterine atrophy, bone loss, and estrogen reduction, all of which commonly occur in postmenopausal women; therefore, they have been widely used to evaluate the estrogenic activity of natural compounds (24). The administration of HRT, which comprises a mixture of estrogen and progesterone, is used to treat menopausal symptoms. However, the chronic use of hormone therapy is associated with a high risk of various cancers (including breast, colorectal, and endometrial) and other serious side effects, such as irregular vaginal bleeding, weight gain, abdominal bloating, and breast pain (37). Consequently, phytoestrogens have emerged as a safer alternative for managing the symptoms of menopause (10). Studies on mitigating menopausal symptoms using phytotherapeutic agents with high bioavailability and few side effects are currently being conducted. In this study, we investigated the phytoestrogenic activity of KOK and the KOK + *P. lobata* mixture against postmenopausal symptoms, specifically postmenopausal osteoporosis.

The effect of ovariectomy was clearly observed in the OVX rats (OVX, OP, OK, OKL, and OKH groups), in which E2 hormone levels, serum lipid levels, bone biomarkers, body weight, tail temperature, endometrial thickness, and uterus weight were significantly altered compared to those in the Sham group. However, the KOK and KOK + *P. lobata* mixture treatments inhibited uterus weight loss, body weight gain, and tail temperature increase and increased the endometrial thickness in the OK, OKL, and OKH groups. These results suggested that KOK and KOK + *P. lobata* mixture can treat postmenopausal symptoms in OVX rats.

Body weight and body weight gain were significantly higher in OVX rats than in the Sham group rats, which may be related to estrogen insufficiency. The effect of estrogen insufficiency on lipid metabolism during menopause has been previously investigated (38), and it is the main cause for the increase in adiposity, particularly abdominal fat accumulation (39). However, by observing the body weight change, it was confirmed that the supplementation of KOK and KOK + *P. lobata* resulted in reduced body weight gain from the 8^th^ week (Figure 2), and eventually, the body weight gain decreased in a dose-dependent manner.

The tail temperature of the experimental animals was measured to verify the palliative effect on hot flushes, a representative symptom of menopause. The rat tail is important for dissipating heat and is the most commonly used site for temperature measurements (40). Based on measurements from week 1 to 7 since the beginning of oral administration, the tail temperature of both the KOK and KOK + *P. lobata* supplemented groups significantly decreased compared to that of the OVX group. In previous studies, the increase in tail skin temperature was observed from the 1^st^ week after ovarian resection (32). In this study, the tail temperature at week 12 in the OVX group significantly increased by 2.3°C compared to that of the Sham group. Indeed, flushing was demonstrated to occur as a physical phenotype due to ovariectomy. The tail temperature decreased compared to that of the OVX group from the 7th week onwards in both groups (KOK and KOK + *P. lobata* supplemented groups). KOK and KOK + *P. lobata* supplementation resulted in a decrease in tail temperature in this experiment and, according to previous studies, could help improve motor and vascular symptoms, such as facial flushing (41).

Cardiovascular disease and obesity are chronic symptoms of menopause. Postmenopausal women have significant increases in body fat compared to premenopausal women because the main supplier of female hormones changes from the ovaries to adipose tissue (42). As hormones are involved in various metabolic processes of the body as well as reproductive functions, body fat increases after menopause to compensate for the decrease in estrogen levels in the blood (43). Aging and menopause not only alter body fat distribution but are also associated with an increased incidence of abdominal obesity. In addition, menopause causes an increase in TG, TC, LDL-C, and VLDL-C levels and a decrease in HDL-C levels (42, 43). In this study, the serum levels of TG, TC, LDL-C, and VLDL-C were lower in the KOK and KOK + *P. lobata* groups than those in the OVX group, while the HDL-C level was higher in the KOK and KOK + *P. lobata* groups than in the OVX group. All of the serum lipid indicators related to fat accumulation showed significant alleviating effects compared to those in the OVX group, which confirmed that the administration of KOK or KOK + *P. lobata* inhibited serum lipid accumulation. However, no dose-dependent effects were observed for lipid accumulation; thus, further studies on dose determination are needed.

Supplementation of KOK and KOK + *P. lobata* at up to 400 mg/kg did not alter the AST and ALT levels, which are biomarkers of hepatic injury. This result is similar to that of a previous study, which reported that KOK and *P. lobata* are not highly toxic based on a toxicity assessment (17). These data suggest that 12 weeks of continuous intake of KOK and KOK + *P. lobata* mixture at this dose did not cause any adverse effects in the liver of OVX rats. We also tested the serum E2 levels of all the groups to confirm if KOK and KOK + *P. lobata* affected the most representative hormone, estrogen. After menopause and ovariectomy, estrogen is not secreted by the ovaries; therefore, the serum E2 levels decrease. However, abnormally high serum E2 levels pose a risk of breast and endometrial cancer, and cardiovascular disease (44). Considering the change in E2 concentration according to the ovulation cycle in previous studies (35), it was confirmed that the administration of KOK and *P. lobata* in this experiment did not affect the E2 levels.

The prevalence of osteoporosis increases with age, and bone loss is reportedly more rapid in females in the first few years after menopause, influenced by estrogen deficiency (45). Bones maintain their density and microstructure through bone remodeling, which involves repeated bone absorption by osteoclasts and bone formation by osteoblasts. Estrogen plays an important role in this process, and osteoporosis occurs after menopause due to this decrease in estrogen levels (46). When estrogen levels decrease, many osteoblasts are produced, and accordingly, the bone turnover rate increases rapidly (45, 46). In this study, we confirmed that KOK and KOK + *P. lobata* supplementation elevated the BMD and the serum osteocalcin levels in OVX rats. Osteocalcin is essential for osteoblast activation and can alleviate postmenopausal osteoporosis by balancing the osteoblast-to-osteoclast ratio. These results imply that KOK and KOK + *P. lobata* exert anti-osteoporotic effects by maintaining the balance between bone resorption and formation.

Estrogen receptors include ER-α and ER-β, which are key receptors for maintaining ovarian granulocyte differentiation, follicle and oocyte growth and development, and ovulation function (47). ER-α is mainly related to and expressed in genital organs such as the uterus and breast, whereas ER-β is mainly related to bone function and expressed in many tissues such as the central nervous system, cardiovascular system, immune system, reproductive system, digestive system, kidneys, and lungs (48). In women, the proportion of these two receptors is balanced before menopause (49). However, if these fail to function normally after menopause and mutations in DNA occur, such as intragenic polymorphisms in ER-α and ER-β, ER function can be affected and chronic diseases may develop (46, 47). Until recently, the most widely known mutations were PvuII and Xbal, which are linked to breast cancer, ovarian cancer, endometrial cancer, uterine myoma, cardiovascular disease, and several other diseases (47). Accordingly, previous studies have reported that *in vitro* estrogen administration has a meaningful protection effect after menopause (50, 51). A meta-analysis has also shown that estrogen administration significantly reduces the risk of cardiovascular disease (52). However, in randomized controlled clinical trials designed to demonstrate the disease prevention effect of estrogen administration, it failed to reduce the incidence of menopause-related diseases, and rather increased the risk of cardiovascular disease and breast cancer (53, 54). Thus, the expression of ER-α and ER-β was an important indicator to determine whether the KOK and KOK + *P. lobata* mixture could be a promising alternative to HRT to alleviate menopausal symptoms. HRT, which is currently used as a treatment for menopausal symptoms including postmenopausal osteoporosis, has a risk for breast cancer and cardiovascular disease by using estrogen and progesterone (10). Estrogen plays a central role in the development and growth of normal and malignant mammary gland tissue, and ER-α mediates most of the action. On the other hand, several *in vitro* experiments have shown that ER-β inhibits the proliferation, migration, and invasion of BC cells and the angiogenesis and growth of tumor xenografts (55–57). In this context, the ER-alpha and ER-beta ratio in this study mean that the smaller the value, the lower the risk for estrogen-induced diseases such as breast cancer. In this study, the ER-α/ER-β ratio was reported to be at the same level as that of the Sham and OP groups. The ER-α/ER-β ratios of the groups OK, OKL, and OKH were relatively low and similar to that of the OVX group. Accordingly, this study confirmed that the administration of KOK and KOK *+ P. lobata* mixture for 12 weeks did not increase the risk of ER activity, potentially decreasing the risk of female diseases such as breast cancer and uterine myoma.

Autophagy is a catabolic pathway by which cell components are degraded to maintain their essential activity and has a cellular protective effect on cell growth, survival, proliferation, and differentiation. Genetic studies have shown that ATG1/ULK1 kinase plays a vital role in the induction of autophagy. Autophagy is promoted by AMPK, a key energy sensor that regulates cellular metabolism and maintains energy homeostasis. Conversely, autophagy is inhibited by mTOR, a central cell-growth regulator that integrates growth factor and nutrient signals (58, 59). We confirmed that the KOK and *P. lobata* extracts effectively alleviated postmenopausal osteoporosis by regulating autophagy, which was related to the activation of AMPK and ULK1 by ginsenoside and honey in KOK, and regulation of mTOR expression by puerarin in *P. lobata*. The phytoestrogenic efficacy of KOK and KOK + *P. lobata* mixture on autophagy induced suppressive effects on osteoblast differentiation and mineralization, thereby alleviating postmenopausal osteoporosis in OVX-induced rats. The phosphorylating AMPK expression did not change significantly among any groups; however, the groups administrated KOK and KOK + *P. lobata* mixtures showed an increase in the phosphorylated ATG1/ULK1 expression and a decrease in the phosphorylated mTOR expression compared with those of the OVX group. The degree to which the ULK1 levels increased and the mTOR levels decreased in these groups was higher than that in the OP group. Subsequently, the phosphorylation of ULK1 was significantly higher in the OK, OKL, and OKH groups than in the OVX group, whereas the phosphorylation of mTOR was significantly lower. In addition, without activating ER-α, which causes female diseases such as breast cancer and uterine myoma, ER-β activated AMPK in the liver, the center of energy metabolism and autophagy, causing inhibition of mTOR, an indicator of aging and autophagy. This is thought to have a positive effect on the alleviation of menopausal osteoporosis through autophagy.

Because this is the first *in vivo* study of KOK and the *P. lobata* mixture, further studies are needed to elucidate the detailed mechanisms by which each component of KOK and KOK + *P. lobata* extracts alleviate postmenopausal osteoporosis. Additionally, because we only assessed the effects of KOK and KOK + *P. lobata* at three dosages (OK: KOK 300 mg/kg, OKL: KOK 300 mg/kg + *P. lobata* 50 mg/kg, OKH: KOK 300 mg/kg + *P. lobata* 50 mg/kg) in a rat model, further studies are needed to determine the optimal dosage for menopausal women. Based on our promising results, KOK and the KOK + *P. lobata* mixture are candidate therapeutic agents against postmenopausal osteoporosis that we expect to see in clinical applications following further research in the menopausal female population.

## Conclusion

The present study demonstrated that KOK and KOK + *P. lobata* mixture showed phytoestrogenic activity, indicating their anti-osteoporosis efficacy. Supplementation with KOK and KOK + *P. lobata* mixture did not affect liver function, the estradiol levels, and inhibited weight gain, a tail temperature increase, and lipid accumulation. Furthermore, the strengthening effects of KOK and KOK + *P. lobata* mixture on bone markers and endometrial thickness were dose-dependent. These results suggest that KOK and the KOK + *P. lobata* mixture are safe alternatives to traditional treatments for postmenopausal osteoporosis and other menopausal symptoms; furthermore, this phytoestrogen-activating mixture may induce synergistic pharmacological effects.

## Data availability statement

The datasets presented in this study can be found in online repositories. The names of the repository/repositories and accession number(s) can be found in the article/Supplementary material.

## Ethics statement

The animal study was reviewed and approved by Animal Ethics Committee of the Sookmyung Women’s University for the Care and Use of Laboratory Animals (SMWU-IACUC-2102126).

## Author contributions

MK and H-SK contributed to conception and design of the study. YK, H-JK, and JJ standardized and provided the sample materials. MK performed the *in vivo* study. JO, XZ, and SA supported the *in vivo* study. MK conducted overall experiments, organized the database, statistical analysis, and wrote the original draft of the manuscript. All authors contributed to manuscript revision, read, and approved the submitted version.

## Conflict of interest

YK, H-JK, and JJ were employed by Kwangdong Pharm Co. Ltd.

The authors declare that this study received funding from Kwangdong Pharm Co. Ltd. The funder had the following involvement in the study: study design, investigation, and the decision to submit it for publication.

## Publisher’s note

All claims expressed in this article are solely those of the authors and do not necessarily represent those of their affiliated organizations, or those of the publisher, the editors and the reviewers. Any product that may be evaluated in this article, or claim that may be made by its manufacturer, is not guaranteed or endorsed by the publisher.

## Abbreviations

ALT: alanine aminotransferase
AMPK: AMP-activated protein kinase
ARRIVE: Animal Research: Reporting of *In Vivo* Experiments
AST: aminotransferase
ATG1: autophagy-related gene 1
BMD: bone mineral density
DEXA: dual energy X-ray absorptiometry
DNA: deoxyribonucleic acid
E2: estradiol
ER: estrogen receptor
H&E: hematoxylin and eosin
HDL-C: high-density lipoprotein cholesterol
HRT: hormone replacement therapy
KOK: Kyung-Ok-Ko
LDL-C: low-density lipoprotein cholesterol
LD50: lethal dose 50
MHF: menopausal hot flush
mTOR: Mammalian target of rapamycin
OVX: ovariectomize
*P. lobata*: *Pueraria lobata* Ohwi
SDS-PAGE sodium dodecyl sulfate polyacrylamide gel electrophoresis
SEM: standard error of mean
TC: total cholesterol
TG: triglyceride
ULK1: Unc-51-like kinase 1
VLDL-C: Very low-density lipoprotein cholesterol
