# Supplementary material for: Ameliorative effects of Kyung-Ok-Ko and its mixture with Pueraria lobata Ohwi on postmenopausal osteoporosis by promoting phytoestrogenic activity in rats
Source: Front Nutr. 2023 Jun 26;10:1171346. doi: 10.3389/fnut.2023.1171346 (PMC10332514; doi:10.3389/fnut.2023.1171346)
Supplement: Supplementary file 1 [file Table_1.docx]

**Supplementary table S1. The antibodies for Western blot**

| **Name** | **Company** | **Product Number** | **Source** | **MW(kDa)** | **Dilution** |
| --- | --- | --- | --- | --- | --- |
| ER-α | Cell Signaling | #8644 | Rabbit IgG | 66 | 1:1000 |
| ER-β | Cell Signaling | #5513 | Rabbit | 55 | 1:1000 |
| GAPDH | Cell Signaling | #5174S | Rabbit IgG | 37 | 1:5000 |
| AMPKa | Cell Signaling | #2532S | Rabbit | 62 | 1:500 |
| p-AMPKa | Cell Signaling | #2531S | Rabbit | 62 | 1:1000 |
| mTOR | Cell Signaling | #2972S | Rabbit | 289 | 1:1000 |
| p-mTOR | Cell Signaling | #2971S | Rabbit | 289 | 1:1000 |
| ULK1 | Cell Signaling | #8054S | Rabbit IgG | 150 | 1:1000 |
| p-ULK1 | Cell Signaling | #14202S | Rabbit IgG | 150 | 1:1000 |
